# Obesity management for the treatment of type 2 diabetes: emerging evidence and therapeutic approaches

**DOI:** 10.3389/jpps.2024.13065

**Published:** 2024-06-06

**Authors:** Arianne Morissette, Erin E. Mulvihill

**Affiliations:** ^1^ The University of Ottawa Heart Institute, Ottawa, ON, Canada; ^2^ Department of Biochemistry, Microbiology and Immunology, The University of Ottawa, Faculty of Medicine, Ottawa, ON, Canada

**Keywords:** type 2 diabetes mellitus, gut hormones, glucagon like peptide 1, metabolic surgery, obesity

## Abstract

Excess adiposity can contribute to metabolic complications, such as type 2 diabetes mellitus (T2DM), which poses a significant global health burden. Traditionally viewed as a chronic and irreversible condition, T2DM management has evolved and new approaches emphasizing reversal and remission are emerging. Bariatric surgery demonstrates significant improvements in body weight and glucose homeostasis. However, its complexity limits widespread implementation as a population-wide intervention. The identification of glucagon-like peptide 1 (GLP-1) and the development of GLP-1 receptor agonists (GLP-1RAs) have improved T2DM management and offer promising outcomes in terms of weight loss. Innovative treatment approaches combining GLP-1RA with other gut and pancreatic-derived hormone receptor agonists, such as glucose-dependant insulinotropic peptide (GIP) and glucagon (GCG) receptor agonists, or coadministered with amylin analogues, are demonstrating enhanced efficacy in both weight loss and glycemic control. This review aims to explore the benefits of bariatric surgery and emerging pharmacological therapies such as GLP-1RAs, and dual and triple agonists in managing obesity and T2DM while highlighting the caveats and evolving landscape of treatment options.

## Introduction

Obesity represents a multifaceted, chronic condition characterized by an accumulation of excessive body fat, known as adiposity, which can impair health and decrease lifespan [1]. Epidemiologic studies define obesity using the body mass index (BMI), which can stratify obesity-related health risks at the population level. Obesity is clinically defined as a BMI exceeding 30 kg/m^2^ and is subdivided further into class 1 (30–34.9 kg/m^2^), class 2 (35–39.9 kg/m^2^) and class 3 (≥40 kg/m^2^). At the population level, complications from obesity rise as BMI increases [2]. At the individual level, the relationship between health complications and BMI is influenced by diverse factors such as the extent of adiposity, its distribution throughout the body, and an array of environmental, genetic, biological, and socioeconomic influences [3]. Excessive adiposity can predispose to metabolic complications, such as type 2 diabetes mellitus (T2DM) [4]. T2DM is defined by hyperglycemia resulting from tissue insulin resistance and relative insulin deficiency [4]. Estimates indicate that approximately 537 million individuals worldwide had T2DM in 2021, a figure that is expected to increase by 46%–783 million by 2045 [5]. Individuals with T2DM are at high risk for microvascular complications, including retinopathy, nephropathy and neuropathy, and macrovascular complications such as cardiovascular comorbidities [6].

For years, T2DM has been viewed as a chronic, progressive condition necessitating continual adjustment of pharmacotherapy, with estimates that 50% of patients will require insulin dependence within 9–10 years [7]. However, a growing body of research challenges this timeline by introducing surgical and pharmacotherapy approaches to managing the disease, emphasizing reversal and remission [8]. Indeed, sustained weight loss of at least 15% of body weight has a positive effect on the progression of T2DM, inducing remission in a large proportion of patients and markedly improving metabolic status in many others [9, 10]. The World Health Organization now openly acknowledges that a window of time exists in which T2DM is metabolically reversible - which is defined as a normal HbA1c without glucose-lowering medications for at least 3 months [11]. Pioneering work by Pories et al. [12] laid the foundation for the notion that bariatric surgery could effectively address T2DM owing to its substantial impact on weight reduction and significant improvements in blood glucose levels, fasting insulin, and HbA1c. Subsequent studies have consistently reaffirmed the efficacy of bariatric surgery in enhancing glucose homeostasis, diminishing the requirement for glucose-lowering medications, and mitigating both microvascular and macrovascular complications associated with T2DM [13]. Notably, some patients have experienced complete remission of T2DM following surgery [13]. Furthermore, evidence suggests that individuals undergoing bariatric surgery are significantly less likely to receive a diagnosis of T2DM even 15 years post-surgery compared to those who do not undergo the procedure [14]. Despite its considerable benefits, a complex surgical procedure is not feasible or scalable as the mainstay for a population-wide intervention.

**FIGURE 1:**
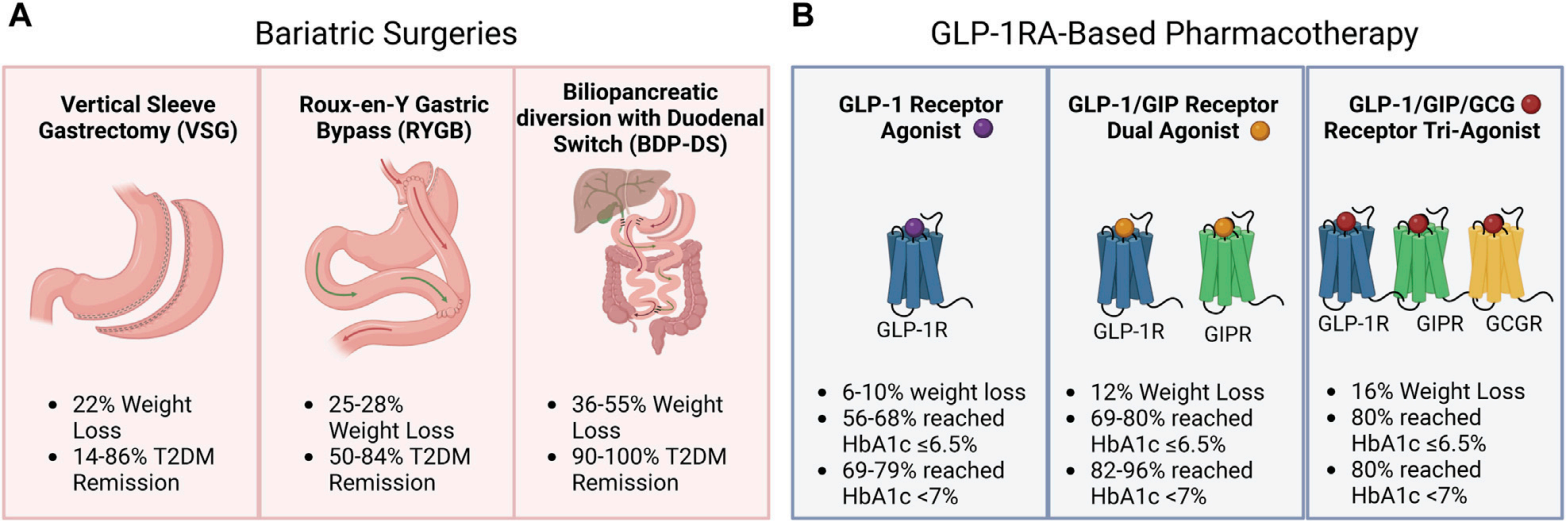
Weight loss and type 2 diabetes mellitus (T2DM) management with bariatric surgery and Glucagon-like peptide 1 receptor agonist (GLP-1RA)-based pharmacotherapy. **(A)** Weight loss (%) and type 2 diabetes mellitus (T2DM) remission (%) were observed following vertical sleeve gastrectomy, Roux-en-Y Gastric Bypass, or biliopancreatic diversion with duodenal switch. **(B)** Weight loss (%) and HbA1c targets of 6.5% and 7% reached by patients following GLP-1RA-based pharmacotherapy.

The discovery that glucagon-like peptide-1 (GLP-1) enhances insulin secretion in a glucose-dependent manner and suppresses glucagon release while minimizing the risk of hypoglycemia has led to the development of various structurally distinct GLP-1 receptor (GLP-1R) agonists (GLP-1RAs) with longer circulation times for the management of T2DM [15–17]. Beyond their now well-defined role in managing glucose levels, GLP-1RAs have emerged as important tools in weight management strategies for individuals living with obesity and T2DM. This effect on body weight primarily stems from their ability to reduce food intake and slow gastric emptying [18]. Innovative treatment approaches combining GLP-1RAs with other gut hormone-derived agonists, such as glucose-dependent insulinotropic polypeptide (GIP), and pancreatic hormone-derived agonists, such as glucagon (GCG) and amylin, are demonstrating promising outcomes, further enhancing both weight loss and glycemic control [19, 20]. This new line of pharmaceuticals to reduce body weight and decrease glucose levels could therefore be a more accessible treatment alternative for individuals living with obesity and T2DM (Figure 1).

This review aims to explore bariatric surgery, currently considered the most effective intervention for addressing obesity and T2DM, and the potential pharmacological emerging therapies such as GLP-1 receptor agonists (GLP-1RAs), dual agonists, and tri-agonists in body weight and T2DM management. Additionally, we will discuss the caveats and potential future directions in treating patients living with obesity and T2DM.

## Bariatric surgery

Bariatric surgery, also known as metabolic surgery, is an effective therapy that helps people with severe obesity achieve significant weight loss while decreasing related cardiometabolic comorbidities [21, 22]. The term metabolic surgery acknowledges the physiological changes caused by the procedure, which leads to a more favourable metabolic profile beyond the traditional belief that it is only provided through weight-dependant mechanisms [23–25]. The most widely performed bariatric surgeries are vertical sleeve gastrectomy (VSG), which consists of removing ∼80% of the stomach along the greater curvature, and Roux-en-Y gastric bypass (RYGB), which involves gastric size restriction with the creation of a small gastric pouch and re-routing of the intestinal tract, such that ingested nutrients empty directly into the jejunum and bypass 95% of the stomach, duodenum and proximal jejunum [26]. Biliopancreatic diversion with duodenal switch (BPD-DS) is a less-common procedure consisting of a sleeve gastrectomy followed by re-routing of the small intestine so that the ileum now connects to the pylorus of the stomach, bypassing both the jejunum and the duodenum [27].

Studies have shown that patients living with T2DM undergoing BDP-DS tend to lose between 36% and 55% of their initial body weight after 10 and 3 years, respectively [28, 29], compared to 28% with RYBG [30] and 22% with sleeve gastrectomy after 10 years [30]. Similarly, BPD-DS is the procedure conferring the highest rate of long-term (2–5 years) diabetes remission, ranging from 90 to 100% [27, 31] compared to 50–84% [29, 32] for RYGB and 14–86% for sleeve gastrectomy [33–35]. The longer duration of diabetes and the type of antidiabetic therapy used before surgery could influence postsurgical glycemic outcomes, thus explaining the heterogeneity in diabetes remission following bariatric surgery [36–38]. Despite being recognized for its durability in terms of weight loss and diabetes remission [29, 39, 40], DPB-DS constitutes only 2.2% of bariatric surgeries performed worldwide [41]. The technical complexity and demanding post-operative monitoring needed to avoid malnutrition due to the malabsorptive nature of this surgery may explain the reduced surgeries employing BPD-DS. As it is a more straightforward procedure that requires a shorter operative time, VSG is now the most widely performed bariatric surgery worldwide [21].

The precise mechanisms resulting in improved glucose control following bariatric surgery remain unclear. The degree of weight loss achieved is generally associated with the degree of resolution of T2DM [9, 42, 43], suggesting that those with greater weight loss after surgery have a greater propensity for improved management of T2DM and remission than those with less weight loss [44]. Indeed, weight loss yields a reduction in total, visceral and pancreatic adipose tissue, reductions in intrahepatic levels of lipids, and improved insulin sensitivity, all of which are expected to improve systemic glucose homeostasis [45]. One study demonstrated that in patients living with obesity and T2DM, 18% weight loss achieved either by RYGB or caloric restriction resulted in similar improvements in insulin sensitivity and β-cell function, suggesting that metabolic improvements are weight-related [44]. Metabolic surgery has also been found to have well-documented effects on improving blood glucose levels [13] and even achieving T2DM remission on a faster timeline that is disassociated from weight loss [21]. These weight-loss-independent improvements are thought to be in part related to changes in bile acid dynamics [46] and microbiota composition [47], a shift in gut physiology, including nutrient intake, gastric emptying, gastric acid production [48], and increases in postprandial gut hormone secretion [49]. Other factors to consider in T2DM remission following bariatric surgery include disease duration, age, and the level of glycemic control [9, 50]. These factors, linked to β-cell functional capacity, suggest that T2DM remission might be more achievable in patients with shorter disease duration, younger age, and better glycemic control. Nevertheless, it was reported in patients with T2DM using insulin before BPD-DS, 97% of patients had cessed insulin therapy after 10 years postoperatively [51].

Overall, the magnitude of change in body weight and glycemic control depends on the type of bariatric surgery performed and the improvements in T2DM management are related to both weight-loss-dependent and independent mechanisms.

## GLP-1RA-based therapies

GLP-1 and GIP are incretin hormones released from gut enteroendocrine cells following a meal and potentiate glucose-dependent insulin secretion from the pancreas [52]. They exert their incretin actions through two distinct yet structurally related class B G protein-coupled receptors, the GIPR and the GLP-1R. These receptors are expressed in several organs tightly controlling energy homeostasis and metabolism, including the pancreas, cardiovascular system, and central and peripheral nervous system [52]. The essential role of incretin receptors in glucose homeostasis was demonstrated in single and double incretin receptor knockout mice. *Glp1r* ^−/−^ mice, and, to a greater extent, *Glp1r* ^−/−^ and *Gipr* ^−/−^ mice, exhibit impaired glucose tolerance and defective insulin secretion when fed a high-fat diet [53]. GLP-1 also exerts anorectic effects by activating GLP-1R + neurons in the hypothalamus and brainstem, which reduces food intake and promotes weight loss [54]. The action of GLP-1 to reduce glycemia by stimulating insulin secretion in a glucose-dependent manner provided the rationale for exploring incretin-based therapies and led to the approval of the first GLP-1R agonist in 2005 for treating T2DM [15]. The use of two GLP-1RAs, liraglutide and semaglutide, for weight loss was later approved in 2014 [55, 56].

The observed reduction in body weight with the use of the GLP-1RA liraglutide (1.2 and 1.8 mg once daily) in individuals living with T2DM prompted the exploration of higher doses of liraglutide in the treatment of overweight and obesity in the Satiety and Clinical Adiposity—Liraglutide Evidence (SCALE) program [55, 57–60]. In the Scale Diabetes trial, an average of 6% weight loss was achieved over 52 weeks in 623 individuals living with T2DM treated with 3 mg liraglutide once daily, with 25,2% of the participants experiencing >10% weight loss. Furthermore, 56.5% of participants receiving 3 mg liraglutide daily achieved a HbA1c ≤ 6.5%, which is considered prediabetic, compared to 15% in the placebo group, and 69.2% reached the target HbA1c <7% set by the American Diabetes Association (ADA) compared to 27.2% in the placebo group [58, 61].

The GLP-1RA semaglutide was also evaluated for the treatment of obesity in the Semaglutide Treatment Effect in People with Obesity (STEP) program at a dose of 2.4 mg once weekly [56, 62–64]. STEP 2 evaluated weight loss in 1,210 individuals living with T2DM and overweight/obesity not treated with insulin (HbA1c 7–10%). Participants were randomized to placebo, semaglutide 1 mg or semaglutide 2.4 mg weekly, together with lifestyle interventions over 68 weeks. Those receiving the highest dose lost an average of 9.6% of their body weight, compared to 3.4% with the placebo. At the highest dose, more than a quarter of the participants lost over 15% of their weight, almost half lost 10%, while two-thirds lost a minimum of 5%. After 68 weeks, participants receiving 2.4 mg had an average HbA1c of 6.4%, in the prediabetic range, and therefore below the threshold to diagnose T2DM, compared to 7.8% in the placebo group. After 68 weeks, 78.5% and 67.5% of those receiving 2.4 mg semaglutide weekly reached the <7% HbA1c target and ≤6.5% prediabetic range, respectively, compared to 26.5% in the placebo group [62].

The efficacy of GLP-1RA in managing body weight and T2DM has spurred significant efforts toward developing next-generation therapies that surpass the effectiveness of GLP-1RA alone. Tirzepatide, a novel dual GLP-1 and GIP analogue, was investigated at weekly subcutaneous doses of 5mg, 10mg and 15 mg compared to 1 mg semaglutide for 40 weeks in patients living with T2DM in the SURPASS phase 3 clinical trial program. The highest tirzepatide dose led to an 11.2 kg (11.9%) weight loss and decreased HbA1c by 2.3%. A total of 82–96% of the patients who received tirzepatide and 79% of those who received semaglutide reached the HbA1c target of <7.0%. Furthermore, HbA1c ≤ 6.5%%, which is considered prediabetic, was met in 69–80% of patients receiving tirzepatide compared to 64% of patients receiving semaglutide [65]. These findings are encouraging, highlighting the promising potential of tirzepatide in the management of T2DM.

Recently, tri-agonists (GLP-1/GIP/GCG) were shown to provide even greater improvements in glycemic control and robust reduction in body weight in individuals living with T2DM. In a phase 2 clinical trial including 281 participants with T2DM and a mean HbA1c of 8.3%, weekly administration of 12 mg retatrutide (starting dose 2 mg) for 36 weeks decreased HbA1c by 2.16% and participants lost ≥15% of body weight compared to baseline. Approximately 80% of those receiving the highest dose of retatrutide reached the <7.0% HbA1c target established by the ADA and roughly the same percentage attained the ≤6.5% HbA1c prediabetic level [66]. These outcomes align with the potential reversal of T2DM [10, 67]. Another study investigating the combination of semaglutide with the long-acting amylin analogue cagrilintide in patients living with T2DM also resulted in significant improvements in body weight and HbA1c in a phase 2 trial. Compared to baseline, once-weekly 2.4 mg of CagriSema for 32 weeks resulted in a 2.2% decrease in HbA1c (mean HbA1c of 6.3%) and a 15.6% body weight loss. Eighty-nine percent of patients achieved the <7% HbA1c target, and 75% had a HbA1c ≤ 6.5% considered in the prediabetic range [68].

While additional studies are required to validate the safety and effectiveness of these newer medications in larger cohorts, GLP-1RA-based pharmacotherapy represents a very promising avenue for managing body weight and T2DM.

## Discussion

Bariatric surgery induces significant weight loss and T2DM remission (Figure 1A). However, there are several contraindications to bariatric surgeries, and not all patients may be eligible. As with any other medical intervention, bariatric surgery poses a health risk, such as postoperative surgical complications, and dumping syndrome, and patients need to be closely monitored for micronutrient deficiencies after the intervention [69]. Furthermore, surgical interventions are difficult to scale to reach everyone who could potentially benefit. It is therefore worth investigating if GLP-1RA-based pharmacotherapy could be a more accessible alternative to bariatric surgery for managing body weight and T2DM (Figure 1B).

Despite their safety and efficacy, individuals may experience adverse side effects using GLP-1R agonists, dual and tri-agonists, such as nausea, vomiting, constipation and diarrhea [70]. Furthermore, GLP-1RAs generally require once-weekly subcutaneous injections [55–60, 62–64]. Orally administered GLP-1RA, such as semaglutide and orforglipron represent another effective therapeutic strategy for managing body weight, blood glucose and other cardiometabolic risk factors [71, 72]. Additionally, an oral formulation for a GLP-1R/GIPR agonist is currently being tested in a phase 2 trial (ClinicalTrials.gov ID NCT06068946). One major limitation to the use of GLP-1RA-based therapies remains its high cost. A recent study even suggested that sleeve gastrectomy was cost-saving compared to semaglutide in the treatment of class II obesity and estimated that a 3-fold decrease in the price of semaglutide was needed to achieve nondominance [73]. Furthermore, long-term obesity and T2DM pharmacotherapy may also be required, as cessation of pharmacological treatment is frequently followed by weight regain, even with continued lifestyle intervention [64, 74]. Nevertheless, GLP-1RA-based pharmacotherapy remains a more accessible alternative than bariatric surgery for managing body weight and T2DM.

While both bariatric surgery and GLP-1RA-based pharmacotherapy represent promising options, surprisingly, few studies have directly compared surgery to pharmacotherapy for glycemic control and glycemic control in patients living with obesity and T2DM. Three studies have reported that RYGB and VSG surpass medical therapy in terms of weight loss, glycemic control and reduction in medical use among patients with T2DM [13, 38, 75]. However, it is important to mention that medical therapy, including various oral anti-hyperglycemic agents, insulin, GLP-1RAs and SGLT2 inhibitors, was heterogeneous across participants. Recent studies using GLP-1/GIP/GCG receptor agonists have demonstrated very promising results. It would therefore be interesting to explore whether these findings could be compared to the outcomes of surgery in regards to both weight loss and glycemic control.

In conclusion, bariatric surgery stands as a highly effective option for managing body weight and T2DM, yielding significant benefits. Yet, its widespread implementation faces scalability challenges, limiting access for many who could potentially benefit. In contrast, GLP-1RAs, and more particularly dual and triple agonists, offer a promising alternative, potentially extending to a larger patient population. Future research is imperative to ensure safety, and efficacy, and optimize treatment options, including decreasing side effects commonly reported by patients. Nevertheless, this novel pharmacotherapy could play a pivotal role in managing body weight, and T2DM, and preventing related micro- and macro-vascular complications.
